# Sex differences in brain homotopic co-activations: a meta-analytic study

**DOI:** 10.1007/s00429-022-02572-0

**Published:** 2022-10-21

**Authors:** Chiara Bonelli, Lorenzo Mancuso, Jordi Manuello, Donato Liloia, Tommaso Costa, Franco Cauda

**Affiliations:** 1 FocusLab, Department of Psychology, University of Turin, Via Giuseppe Verdi 10, 10124 Turin, Italy; 2 Department of Psychology, GCS-fMRI, Koelliker Hospital, University of Turin, Turin, Italy

**Keywords:** Homotopic connectivity, Activation likelihood estimation, Coordinate-based meta-analysis, Hemispheric integration, Lateralization

## Abstract

An element of great interest in functional connectivity is ‘homotopic connectivity’ (HC), namely the connectivity between two mirrored areas of the two hemispheres, mainly mediated by the fibers of the corpus callosum. Despite a long tradition of studying sexual dimorphism in the human brain, to our knowledge only one study has addressed the influence of sex on HC.

We investigated the issue of homotopic co-activations in women and men using a coordinate-based meta-analytic method and data from the BrainMap database. A first unexpected observation was that the database was affected by a sex bias: women-only groups are investigated less often than men-only ones, and they are more often studied in certain domains such as emotion compared to men, and less in cognition. Implementing a series of sampling procedures to equalize the size and proportion of the datasets, our results indicated that females exhibit stronger interhemispheric co-activation than males, suggesting that the female brain is less lateralized and more integrated than that of males. In addition, males appear to show less intense but more extensive co-activation than females. Some local differences also appeared. In particular, it appears that primary motor and perceptual areas are more co-activated in males, in contrast to the opposite trend in the rest of the brain. This argues for a multidimensional view of sex brain differences and suggests that the issue should be approached with more complex models than previously thought.

**Supplementary Information:**

The online version contains supplementary material available at 10.1007/s00429-022-02572-0.

## Introduction

Two properties were suggested to characterize brain connectivity: segregation and integration (Sporns, 2013). Segregation refers to a network organization characterized by clusters of nodes (neurons or areas) more strongly connected between them than with other clusters, promoting functional specialization (Jager and Postma 2003; Sporns 2013). Integration refers to the interaction between specialized brain regions that allows both long-range synchronization and flow of information (Friston 2002; Heuvel & Sporns 2013). The interaction between these two processes defines the pattern of correlation and anticorrelation between resting state networks (Fox et al. 2005a), as well as the functional lateralization of the two hemispheres (Gotts et al. 2013).

A relevant element of FC is homotopic connectivity (HC). HC refers to the (structural or functional) connectivity between two homologous areas of the two hemispheres, mainly mediated by the fibers of the corpus callosum (Goldstein et al. 2021; Mancuso et al. 2019a; Mollink et al. 2019). Crucially, HC can be taken as an index of local interhemispheric integration (Jin et al. 2020). Values of homotopic FC are higher than those of other forms of intra- and interhemispheric FC (Stark et al. 2008), and it correlates with the performance of cognitive tasks, such as visuospatial attention (Gracia-Tabuenca et al. 2018) and executive function (Zhao et al. 2020; Vallesi et al. 2021). Furthermore, altered patterns of homotopic FC were observed in a wide array of pathologic conditions or abnormal states (Shan et al. 2021; Yu et al. 2018; Chen et al. 2016; Guo et al. 2019; Deng et al. 2021; Loomba et al. 2021; Yao et al 2021; Li et al. 2015; Zhao et al. 2017). The presence of functional HC has been also confirmed by numerous meta-analytic studies based on neuroimaging data and by applying different methods (Laird et al. 2011; Toro et al. 2008). Indeed, previous work from our group adopted a meta-analytical framework to evaluate the homotopic co-activation patterns (Mancuso et al. 2019b), confirming that homotopic FC is stronger for primary than associative regions, as observed by others (Stark et al. 2008; Aboitiz and Montiel 2003). However, that study did not address the possible influence of confounding variables such as age and sex.

The study of sex differences in the human brain is of great interest, also and especially to identify mechanisms underlying behavioral differences or to explain the prevalence of some psychiatric and neurological disorders (Salminen et al. 2021). However, data are often ambiguous and their interpretations are contradictory (Wiersch, L. and Weis, S. 2021; McCarthy, M. M. 2016), and different views on the matter are livelily debated (Wiersch and Weis 2021; McCarthy 2016; Joel et al. 2015; Del Giudice 2021; Joel et al. 2020; Grabowska 2017; Zhang et al. 2021; Joel et al. 2018; Joel 2021; Byne et al. 1988; Hirnstein and Hausmann 2021; Williams et al. 2021). More specifically, it has long been discussed if there are any anatomical differences in the corpus callosum between females and males, with most studies suggesting stronger callosal connectivity in females (Schmied et al. 2020; Ardekani et al. 2013; Suganthy et al. 2003; Habib et al. 1991; Luders et al. 2014; Bruner et al. 2012; Kanaan et al. 2012; Shino et al. 2017). Regarding functional HC, Zuo and colleagues tested the local differences between sexes, reporting greater voxel-wise resting-state HC in the posterior cingulate cortex and in the medial and lateral prefrontal cortex in females; on the contrary, males showed greater HC in the cerebellum, parahippocampal gyrus and fusiform gyrus (Zuo et al. 2010).

These findings are suggestive of different mechanisms of segregation and integration in women and men. In fact, results from Ingalhalikar and colleagues (Ingalhalikar et al. 2014) indicate that females exhibit stronger structural inter-hemispheric connectivity than men, unlike males in which the intra-hemispheric connections prevail compared to the other sex. However, other works suggest that such results do not hold if differences in brain volume are taken into account (Hänggi et al. 2014; Martinez et al. 2017). A related issue is that of sexual differences in hemispheric lateralization. If the characterization by Ingalhaikar and colleagues was true, regardless of any brain volume nuisance, we should expect that women had generally less lateralization and thus stronger interhemispherical co-activations than men. In fact, better microstructural organization of the corpus callosum has been associated with more symmetric processing of dichotic stimuli (Friederich et al. 2017), while regions with weaker callosal connections showed more asymmetric activations (Karolis et al. 2019). These observations suggest that stronger callosal connections may lead to less lateralization and thus more likely homotopic co-activations. Some empirical and theoretical accounts support the hypothesis of a lesser lateralization in female subjects (McGlone 1980; Hiscock et al. 1995), but the literature is inconsistent (Voyer et al. 1995; Boles 2005). For instance, there is some evidence for leftward lateralization from behavioural and FC studies in females, which would explain their greater ability in verbal tasks than males (Hjelmervik 2012; Tomasi and Volkow 2012). On the contrary, males would show lateralization to the right consistently with their better performance in visual-spatial tasks (Voyer et al. 1995; Tomasi and Volkow 2012; Wendt and Risberg 1994). However, other studies provide contradicting evidence (Agcaoglu et al. 2015; Liu et al. 2009; Nielsen et al. 2013).

In the present study, we investigated the matter of homotopic co-activations in women and men, employing the meta-analytical method previously presented by our group (Mancuso et al. 2019b). We assessed the meta-analytical homotopic connectivity (MHC) of female and male subjects on a large database of activation studies obtained from the BrainMap database. There is a large body of evidence showing the similarity between co-activations and functional connectivity (Toro et al. 2008; Smith et al. 2009; Laird et al. 2011; Laird et al. 2013; Torta et al. 2013; Cauda et al. 2021); in particular, the MHC technique has previously been shown to produce similar results to the VMHC (Mancuso et al. 2019b). This would suggest that meta-analytic homotopic co-activations could be taken both as an indicator of interhemispheric integration (vs. lateralization) and of functional connectivity. Furthermore, we chose to focus on singular cognitive domains, to investigate if the patterns of interhemispheric integration of the two sexes varie between them. If it is true that males are more lateralized than females, we should expect to find stronger homotopic co-activations in females. However, given the complex and conflicting results of sex neuroscience, we anticipated that results could be not straightforward, with complex local variations in HC between sexes and across cognitive domains.

## Methods

### Data collection

To obtain the activation data to model the homotopic co-activations, the BrainMap (Fox et al. 2005b; Fox and Lancaster 2002; Laird et al. 2005) functional database section was searched (March 2021) using Sleuth (v. 3.0.4; https://www.brainmap.org/sleuth/). BrainMap is an open-access database of published human neuroimaging experiments reporting coordinates of activation in stereotaxic brain space. The experiments are coded with a cognitive taxonomy divided into 5 domains and 52 subdomains, which can be consulted at https://www.brainmap.org/taxonomy/behaviors.html. Two domain-general queries were carried out for both sexes, as:

[Experiments Context is Normal Mapping] AND [Experiments Activation is Activations Only] AND [Subjects Diagnosis Is Normals] AND [Subject Gender is …].

where Subject Gender was set to Female Only and Male Only, respectively. Then, ten domain-specific queries were conducted using the following algorithm:

[Experiments Context is Normal Mapping] AND [Experiments Activation is Activation Only] AND [Subjects Diagnosis is Normals] AND [Experiments Behavioural Domain is …] AND [Subjects Gender is …].

where, as Behavioural Domain, we selected each time one of the five options (i.e., Action, Emotion, Cognition, Interoception, Perception), always including all the available sub-domains for each main domain. Each query was repeated for both sexes.

Subsequently, the list of experiments was carefully screened to verify that the Sleuth output was consistent with the given query (Manuello et al. 2022), that is, that the studies did not include individuals of the opposite sex, or that all the subjects were healthy and were not taking medications. We also decided to exclude those experiments investigating a group of female subjects during their menstrual cycle, as it was indicated that it could influence the fMRI signal (Fernàndez et al. 2003; Hjelmervik et al. 2014; Donishi et al. 2017; Pritschet et al. 2020). Furthermore, hormonal fluctuation during the menstrual cycle also influences functional laterality (Hausmann et al. 2002; Hausmann 2005; Pletzer et al. 2019). See Supplementary Figures S1-S2 for the PRISMA (Page et al. 2021) flow chart summarizing the selection of studies.

### Meta-analytic homotopic connectivity calculation

As a first step, the cerebral cortex was parcellated in couple of homotopic regions. To do so, the 800 volumes functional atlas by Schaefer et al. (2018) was symmetrized, substituting the right hemisphere with a flipped image of the left one. The purpose of such a passage was to obtain symmetrical MHC maps similar to their non-meta-analytical counterpart, the VMHC. The atlas was also converted to Talairach space. We worked in Talairach space to take advantage of the Talairach Daemon client (Lancaster et al. 2000; Lancaster et al. 1997) to label the areas.

**Fig. 1:**
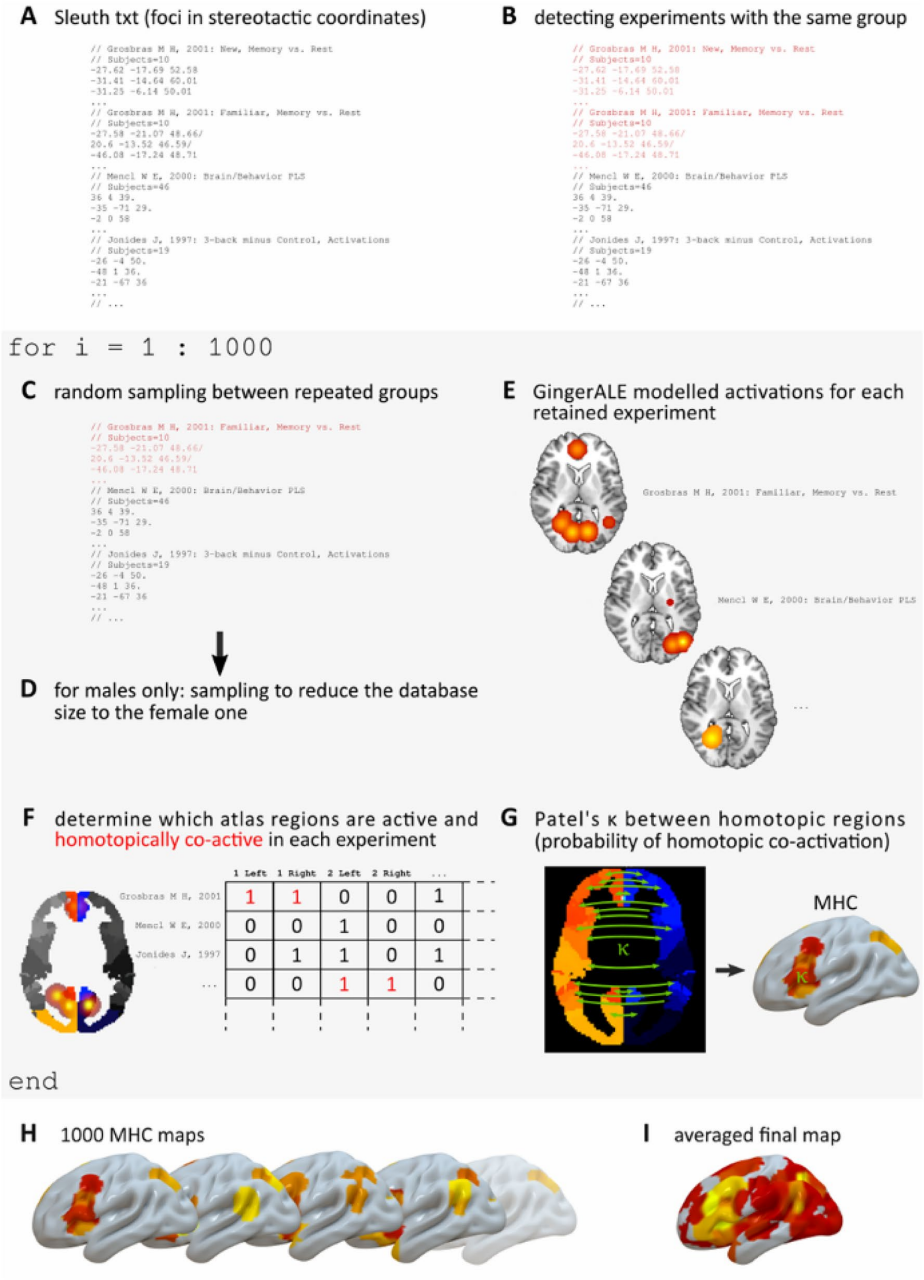
Workflow of the calculation of the MHC map of a given domain in a given sex

Meanwhile, the 12 datasets of coordinates (i.e., 2 domain-general and 10 domain-specific) resulting from the queries were exported from Sleuth in Talairach standard space (Fig. 1A). As pointed out by Müller and colleagues (Müller et al 2018), multiple experiments conducted on the same group of subjects may constitute an invalidation of the requisite of statistical independence between the observations. For this reason, each dataset was scanned to identify such experiments (Fig. 1B). The issue was then addressed with an iterative sampling procedure, with 1000 repetitions. In each iteration, for every set of experiments conducted on the same group of subjects, only one was randomly chosen (Fig. 1C).

The resultant datasets were elaborated as in Mancuso and colleagues (Mancuso et al. 2019b). The activation map of every experiment was reconstructed feeding its list of foci to GingerALE (v. 3.0.2; https://www.brainmap.org/ale/) (Eickhoff et al. 2012; Turkeltaub et al. 2012). In a GingerALE modelled activation map, each focus of activation is used as the center of a 3D Gaussian distribution of probability (Fig. 1E), that estimates the voxel-wise probability of activation of the experiment given its foci (Eickhoff et al. 2009). Such maps are automatically converted to z-scores by the software. To avoid that foci close to the midline could generate unrealistic bilateral activations, we introduced an offset adjustment: If an activation fell in a range of 12 mm from the midline, voxels in the 12 contralateral mm were attenuated in the function of their distance from the midline, with values of more distant voxels being progressively reduced (Mancuso et al. 2019b).

Once obtained the modelled activations for every experiment of the list, each one of the volumes was determined as activated in a given experiment if at least 20% of its voxels were significantly activated, i.e. different from zero in the corresponding modelled activation map, thresholded at *p* = 0.05 (Fig. 1F). The 20% threshold is arbitrary, but we previously showed that different thresholds do not greatly affect the results (Mancuso et al. 2019b). Then, the probability of two homotopic areas to be found co-activated in our sample was computed by means of the Patel’s κ, a Bayesian measure of co-activation, the calculation of which is explained in detail elsewhere (Cauda et al. 2020; Cauda et al. 2021; Patel et al. 2006; Manuello et al. 2018), thresholded at *p* = 0.05. In short, Patel’s κ assesses the probability of a couple of areas of being more co-activated than expected given the observed activations of the two regions, and it is tested for significance with a Monte Carlo simulation. The resulting values were assigned to all the voxels of the two homotopic regions to produce an MHC map (Fig. 1G).

The steps above were repeated 1000 times, each time producing an MHC map that was calculated only with experiments with independent groups of subjects (Fig. 1H). The resulting set of 1000 MHC maps was finally averaged to obtain a map (Fig. 1I) representing the homotopic co-activation of that specific condition. The whole procedure was implemented separately for the 6 female datasets (i.e., 5 behavioural domains plus the domain-general condition, see next subsection for males). To ensure the validity of such maps, the significance of the averages was also tested with SPM12 as one-sample *t* tests, with *p* = 0.05 and FWE correction for multiple comparisons.

### Controlling for females/males imbalance

The BrainMap searches for the male sex returned a greater number of experiments compared to the same searches for females (see Results). The use of a Bayesian method as the Patel’s κ should have prevented issues regarding statistical power, as significance is not directly influenced by sample size in such form of statistics (Costa et al. 2021). However, it may be argued that given the heterogeneity typical of neuroimaging results (Botvinik-Nezer et al*.* 2020) a wider set of experiments, reporting a larger number of foci, might cover a wider portion of the cortex by mere chance. Thus, the comparison between maps obtained from datasets with different sizes might be biased, with a comparatively wider array of co-activations in the map produced with the largest database, because of mere sampling artifacts. More importantly, the domain-general dataset was composed of a different proportion of studies belonging to the different cognitive domains. Thus, the differences between the domain-general maps of the two sexes might have been attributed to their different cognitive composition, with the maps of each sex likely more co-activated in those areas associated with the domains in which they are more often studied.

To overcome such issues, the MHC of males was calculated using an additional sampling passage (Fig. 1D). After the first step in which repeated subject groups were randomly purged from the experiment list of each specific domain, the pool of its experiments was sampled again, reducing its size to match the number of the corresponding female condition. Then, an MHC map was calculated as detailed above, and the procedure was repeated 1000 times. To obtain the male domain-general map, we proceeded in a similar way, sampling its database so each domain subset had the same number of experiments as in the female list. This produced a set of experiments that was as large as the female one and had the same proportion of investigated cognitive functions. These samples were finally averaged and tested for statistical significance as those of women.

### Comparisons between maps

To compare the MHC maps of the two sexes, the average maps of each condition were subtracted as *female*–*male*, so that regions that showed stronger co-activations in the females compared to males had positive values, while the opposite situation resulted in negative values. Such calculation can be seen as a weighted winner-takes-all map, and it was shown in Mancuso et al. (Mancuso et al. 2019b) to be able to highlight statistically relevant divergences. As further validation, the 1000 maps resulting from the sampling procedure for each sex were treated as groups in a two-sample t-test, performed with SPM12 with *p* = 0.05 and FWE correction for multiple comparisons.

## Results

### Search results

The domain-general search resulted in 963 experiments for the female gender and 2199 for the male gender, with 7186 and 18,031 total number of foci and 4796 and 8751 number of subjects, respectively. After randomly sampling them by groups (see “Methods”) we obtained 1000 sets of 234 experiments for the females and 667 experiments for the males. The males dataset was then further sampled to reduce its size to that of the females. Details for the domain-specific searches are provided in Table 1.

**Table 1:** Details for the domain-specific searches. The size of the males datasets indicates the number of experiments obtained by each random sampling to eliminate repeating groups, before further sampling to reduce it to the female size

Behavioral domain–Gender	N. experiments (total)	N. foci (total)	N. subjects (total)	N. experiments after sampling
Action Females	51	623	241	21
Action Males	325	3137	1424	104
Cognition Females	270	2327	1564	95
Cognition Males	1006	8865	4420	333
Emotion Females	240	2092	1260	79
Emotion Males	345	2380	1742	117
Interoception Females	89	604	433	25
Interoception Males	199	2290	946	74
Perception Females	262	1876	1280	81
Perception Males	606	4711	2193	192

**Fig. 2:**
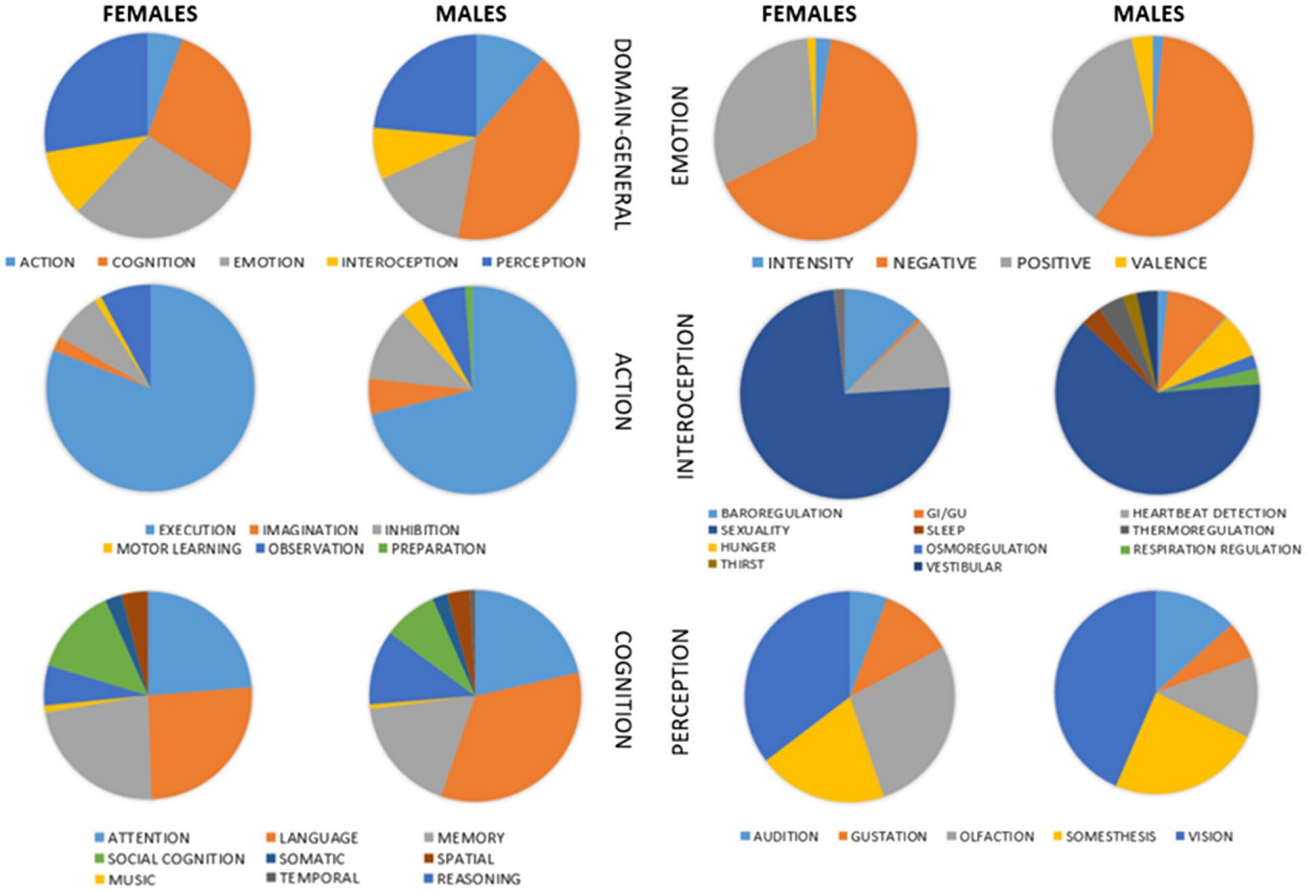
Proportions of investigated cognitive domains and sub-domains by sex. These charts represent the datasets before the random samplings

Importantly, the two domain-general datasets are not balanced in their respective proportions of behavioral domains (Fig. 2). For instance, in both females and males, the most studied category is cognition, but in different proportions (29 vs 42%). Also, the cognitive domains relating to the category of emotions are investigated more in females (28%) than in males (15%). This unbalancement motivated us to implement the second stage of sampling on the males dataset, to match it to the size and proportions of the female one (see “Methods”).

Similarly, there are similarities but also divergences between the sexes within the behavioral domains. With regards to the ‘action’ category, the analysis of executive tasks prevails in both sexes, 81 vs 71% for females and males, respectively. For cognitive tasks, areas concerning language are more investigated in males than females, 34 vs 26%, respectively. Concerning the cognitive domain of emotions, in females more negative emotions were analyzed than in males, 65 vs 58%, respectively. In the context of interoception, sexuality was the most investigated both in females and in males (74 vs 64%, respectively). Finally, the observation of responses to visual stimuli in the ‘perception’ task prevails in both females and males (35 vs 44%, respectively). Supplementary Figure S3 shows further details on behavioral sub-domains. Unfortunately, due to the very small number of experiments in some of these sub-domains, it became unfeasible to match the male datasets to the female ones at the sub-domain level.

**Fig. 3:**
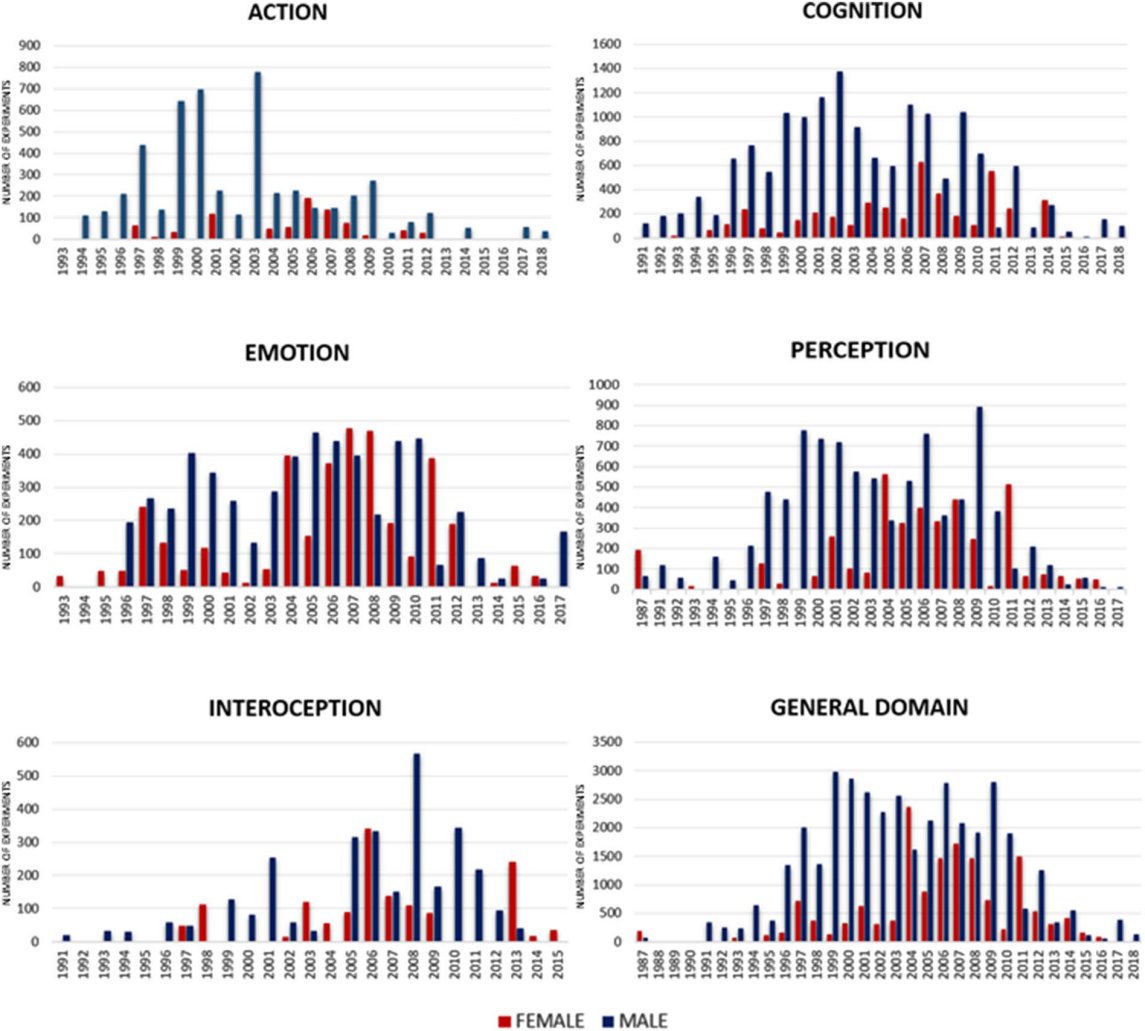
Number of experiments by year for females and males in each cognitive domain

We also investigated if the newer research was at least mitigating those biases. Plotting the number of experiments by year (Fig. 3) showed that after approximately year 2005 there was a substantial increase of female-only experiments in most domains. However, they still remain somewhat in the minority, and the relatively scarce number of post-2010 studies makes it hard to speculate if there is an ongoing tendency in the literature towards balancement. Most importantly, post-2005 pie charts (Supplementary Figure S4) show that the sexes are still investigated preferentially in different domains, despite the increase of female-only studies.

## Meta-analytic homotopic connectivity of the two sexes

**Fig. 4:**
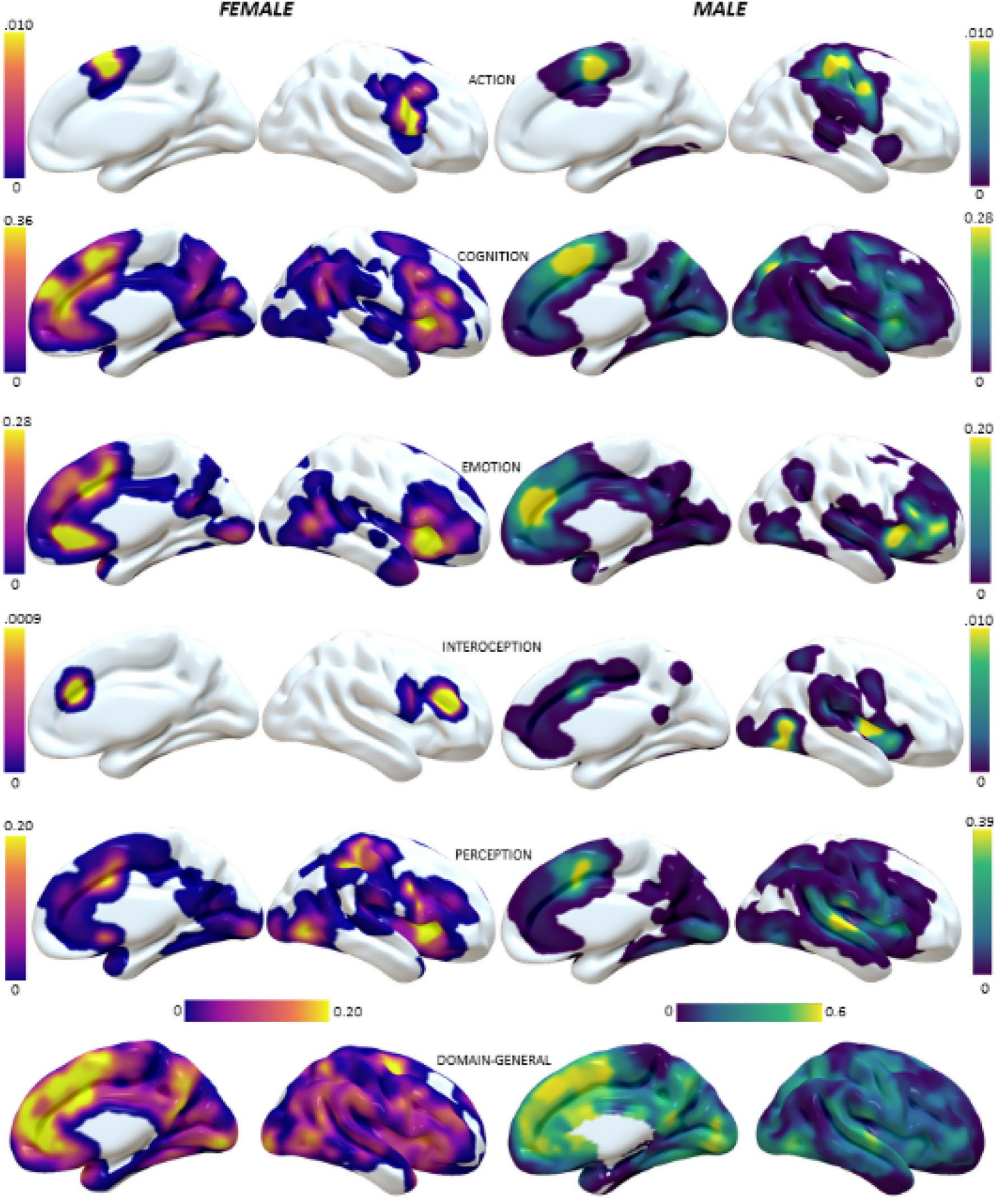
Surface mapping of the averaged MHC maps for the two sexes and the different domains

**Fig. 5:**
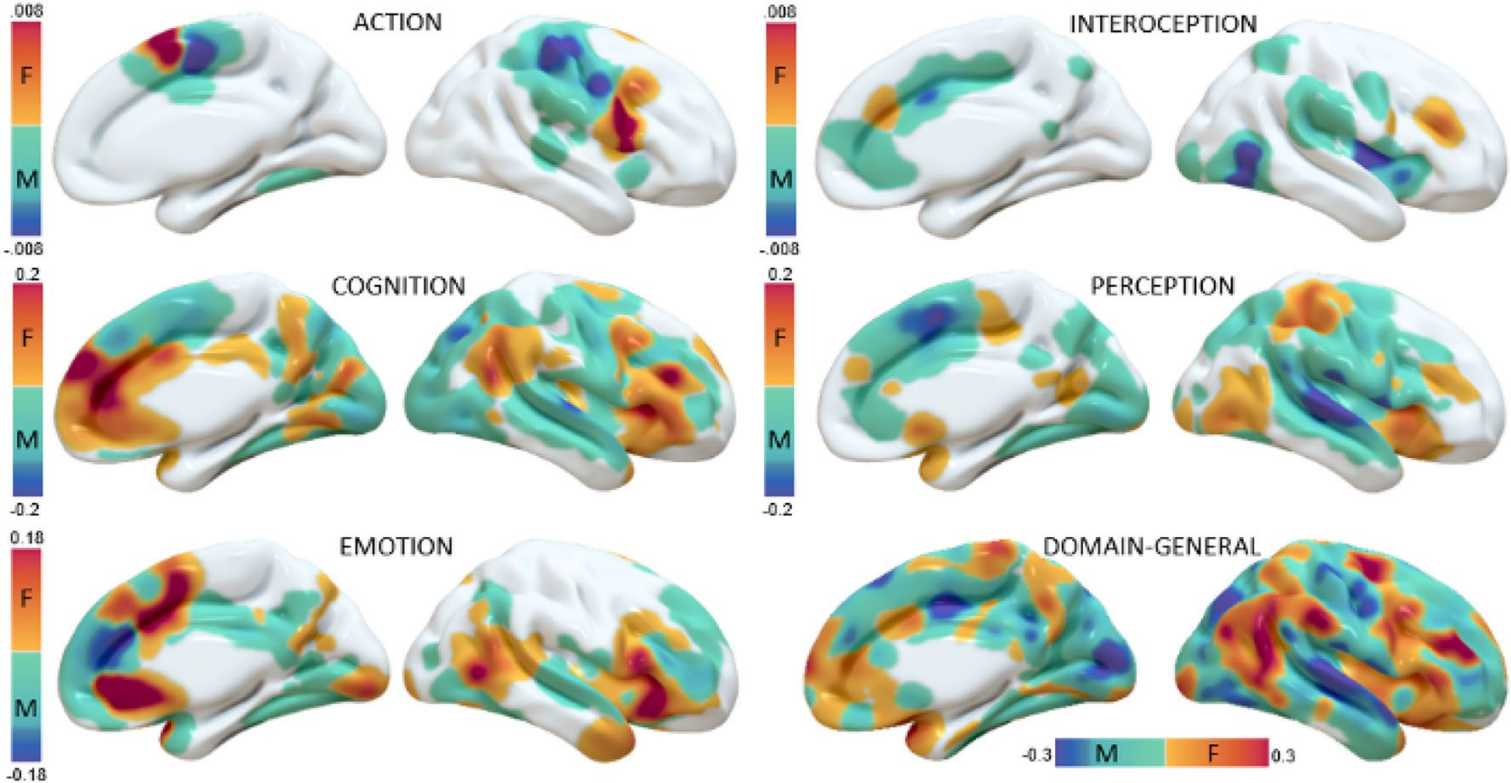
Surface mapping of the differences between the sex in the behavioral domains. Positive values indicate areas more co-activated in females, negative values indicate areas more co-activated in males

The MHC maps of the two sexes show a series of obvious differences between both domains and sexes. Figure 4 shows the average MHC maps, while Fig. 5 illustrates their differences.

The Action domain mainly activates the primary somatomotor cortex in both sexes. However, the peak is in its inferior portion in women, while in males the most active areas are those at the level of the parietal lobe or primary motor cortex. In addition, on the medial surface, the peak of homotopic co-activation in females is anterior to that of males. Cognition tasks co-activate especially the medial and lateral prefrontal cortex (mPFC and lPFC) in both sexes, but females seem to co-activate the ventral mPFC, the insula and the temporo-parietal junction more than males, while males show peaks in the cuneus and superior temporal gyrus (STG). The Emotion domain mostly involves the insula and mPFC, but the first is more engaged in females, while the two sexes show differential homotopic co-activations in different portions of the latter (more dorsal and ventral in females, more central in males). Interoception tasks reveal a marked difference between women and men, with the first co-activating the bilateral middle frontal gyrus (MFG) and a portion of mPFC, and the latter showing higher homotopic co-activations in particular in the posterior insula and the temporo-occipital cortex. Perception tends to co-activate the homotopic areas of a large part of the cortex but the female map is preferentially engaged in areas such as the anterior insula and the superior parietal cortex, while MHC of males peaks in the STG and medial Brodmann Area 6. The domain-general condition shows strong homotopic co-activations in medial cortices in both females and males. However, the two sexes display different landscapes of co-activations. While males’ MHC map shows the typical patterns of homotopic FC with higher values in primary regions compared to associative areas, the same is not so obvious for females. Comparatively, women show more homotopic co-activations than men in TPJ and temporo-occipital cortices, premotor cortices and MFG. Details on the most co-activated homotopic regions in the two sexes for each domain can be found in Table 2. The Jaccard indices calculated for the six couple of maps are presented in Table 3, and confirmed that the Action and Interoception domains show a lesser extent of overlap between significant homotopic co-activations between sexes.

**Table 2:** The ten most co-activated areas of each averaged MHC maps. Brodmann Area (BA) labels were obtained with the Talairach Daemon client. Stereotactic coordinates refer to the left hemisphere volume of a couple of homotopic, symmetrical areas

Domain-general						Action					
Females			Males			Females			Males		
BA	Centroid coordinates	Average value	BA	Centroid coordinates	Average value	BA	Centroid coordinates	Average value	BA	Centroid coordinates	Average value
6	− 29, − 5, 57	0,80	17	− 1, − 78, 10	0,76	44	− 47, 9, 15	0,06	6	− 7, − 4, 51	0,04
24	− 7, 10, 34	0,76	8	− 5, 34, 38	0,73	6	− 48, 4, 22	0,06	4	− 39, − 22, 56	0,02
9	− 5, 52, 27	0,75	6	− 7, − 4, 51	0,71	6	− 7, 7, 53	0,03	6	− 53, − 7, 39	0,02
6	− 22, 1, 51	0,75	8	− 5, 18, 47	0,69	6	− 5, 11, 61	0,03	6	− 46, − 3, 37	0,01
18	− 5, − 83, − 13	0,73	10	− 8, 52, 14	0,68	6	− 55, 3, 20	0,01	3	− 42, − 25, 50	0,01
8	− 5, 34, 38	0,72	24	− 5, 11, 27	0,68	9	− 42, 14, 37	0,01	6	− 5, − 2, 62	0,01
10	− 4, 49, 0	0,71	30	− 5, − 52, 16	0,68	6	− 53, − 7, 39	5	3	− 42, − 17, 47	0,01
6	− 5, − 2, 62	0,70	6	− 5, − 2, 62	0,67	13	− 42, 8, 6	3	40	− 39, − 39, 45	0,01
32	− 7, 39, 11	0,68	18	− 9, − 78, 0	0,67	6	− 5, − 2, 62	1	6	− 5, − 14, 57	0,01
24	− 6, 31, 0	0,68	18	− 3, − 82, 16	0,67	24	− 7, 8, 43	1	6	− 7, 7, 53	0,01

Cognition						Emotion					
Females			Males			Females			Males		
BA	Centroid coordinates	Average value	BA	Centroid coordinates	Average value	BA	Centroid coordinates	Average value	BA	Centroid coordinates	Average value
13	− 31, 19, 4	0,58	6	− 7, 7, 53	0,53	24	− 6, 31, 0	0,70	9	− 7, 41, 26	0,42
9	− 5, 52, 27	0,58	24	− 7, 8, 43	0,48	13	− 34, 26, 6	0,51	13	− 34, 12, − 2	0,41
13	− 40, 22, 8	0,55	8	− 5, 18, 47	0,48	32	− 7, 20, 33	0,40	32	− 9, 30, 27	0,35
6	− 7, 7, 53	0,47	7	− 22, − 65, 41	0,42	47	− 34, 33, − 4	0,39	46	− 47, 27, 19	0,25
6	− 5, 18, 47	0,47	19	− 27, − 58, − 14	0,34	25	− 5, 12, − 4	0,37	32	− 7, 39, 11	0,23
13	− 34, 26, 6	0,44	7	− 11, − 65, 52	0,33	13	− 34, 12, − 2	0,37	8	− 5, 45, 43	0,23
32	− 7, 39, 11	0,43	22	− 53, − 17, 7	0,32	47	− 34, 20, − 7	0,36	13	− 31, 19, 4	0,22
46	− 40, 30, 23	0,41	13	− 42, 8, 6	0,31	24	− 7, 8, 43	0,35	10	− 40, 45, 6	0,21
9	− 7, 41, 26	0,40	13	− 31, 19, 4	0,29	13	− 31, 19, 4	0,33	13	− 34, 26, 6	0,20
24	− 7, 10, 34	0,40	7	− 28, − 58, 45	0,29	13	− 33, 14, 12	0,33	47	− 43, 29, − 3	0,19

Interoception						Perception					
Females			Males			Females			Males		
BA	Centroid coordinates	Average value	BA	Centroid coordinates	Average value	BA	Centroid coordinates	Average value	BA	Centroid coordinates	Average value
46	− 40, 30, 23	0,006	19	− 44, − 68, − 8	0,03	13	− 33, 14, 12	0,60	40	− 57, − 20, 18	0,58
32	− 9, 30, 27	0,004	13	− 38, − 4, 5	0,03	13	− 31, 19, 4	0,48	22	− 53, − 17, 7	0,56
9	− 48, 4, 22	0,001	39	− 46, − 60, 9	0,02	13	− 40, 22, 8	0,42	32	− 7, 8, 43	0,49
			13	− 34, − 3, 14	0,02	40	− 39, − 39, 45	0,39	41	− 57, − 27, 10	0,45
			13	− 42, 8, 6	0,01	40	− 39, − 37, 38	0,34	13	− 49, − 14, 13	0,45
			24	− 7, 10, 34	0,01	40	− 35, − 45, 48	0,33	13	− 47, 0, 7	0,41
			13	− 34, 12, − 2	0,01	6	− 48, 2, 29	0,33	6	− 7, 7, 53	0,41
			37	− 47, − 63, − 1	0,01	13	− 36, 5, 11	0,32	13	− 42, 8, 6	0,41
			13	− 51, − 33, 20	0,01	25	− 5, 12, − 4	0,30	13	− 36, 5, 11	0,38
			13	− 44,− 24, 18	0,01	3	− 26, − 33, 51	0,28	24	− 7, 10, 34	0,38

**Table 3:** Jaccard indices between female and male maps of each domain

	Jaccard index
Domain-general	0.73
Action	0.09
Cognition	0.44
Emotion	0.39
Interoception	0.02
Perception	0.46

A series of one-sample *t* tests (*p* = 0.05, FWE corrected) supported the results of the averaged maps, with the exception of the female Interoception map, which showed no significantly co-activated areas (Supplementary Figure S5). Similarly, a series of two-sample *t* tests confirmed the results of the *female*–*male* differences (Supplementary Figure S6).

In general, males seemed to show more widespread homotopic co-activations than females, although many of those areas had relatively weak values. Conversely, with the exception of the Interoception and Perception maps, females showed larger regions of strong homotopic co-activation. In fact, the number of non-zero voxels was always smaller in the female than the male maps. Also, the average of their values was higher in the female maps, with the exception of Interoception and Perception ones (Table 4).

**Table 4:** Number of non-zero voxels and average value of non-zero voxels for each of the maps presented in Fig. 2, and the ratio between female maps and male maps. Ratios > 1 indicate that the female map has a larger value than that of men, ratios < 1 indicate the opposite

Cognitive Domain	N. non-zero voxels				Average value of non-zero voxels			
	F	M	F/M	*p*	F	M	F/M	*p*
Domain-general	77,226	103,836	0.74	≪ .001	0.29	0.24	1.19	≪ .001
Action	2654	13,328	0.2	.0088	0.02	0.01	4.33	≪ .001
Cognition	41,370	85,480	0.48	≪ .001	0.14	0.07	1.95	≪ .001
Emotion	30,704	43,694	0.7	≪ .001	0.11	0.05	2.14	≪ .001
Interoception	1138	14,084	0.08	≪ .001	.0039	.0041	0.93	.653
Perception	39,278	65,384	0.6	≪ .001	0.07	0.08	0.87	≪ .001

To verify these findings, we calculated these two parameters in each of the 1000 MHC maps used to build the final average maps. So, we performed a series of two-sample, two-tailed t-tests on the difference of these parameters between sex for each domain. All tests were significant at the Bonferroni corrected threshold *α* = 0.008, except for the number of non-zero voxels between the Action maps, which was still very close to significance, and the mean of non-zero voxels of the Interoception domain, which was non-significant. We further tested the significance of these two parameters building a null model based on some sort of sex-neutral brains, obtained averaging maps sampled from both the female and male pools of MHC maps. Methodological details and results are provided in the Supplementary Materials (Table S1). Such tests confirmed that all ratios were highly higher or lower than 1, with the exception of that of the average value of non-zero voxels in the Interoception domain. Furthermore, we tested the hypothesis of a sex imbalance in the distribution of homotopic co-activation with a chi-square test. A 6✕2 (domain-by-sex) matrix has been built (Supplementary Table S2), using our difference maps as input. For each domain, if an atlas area had stronger female co-activations (*F–M* > 0), it was counted in the female column, otherwise (*F–M* < 0) it was assigned to men. Regions whose difference was 0 were discarded. The chi-square test was highly significant (*Χ*^*2*^ = 34.22; *p* ≪ 0.01), indicating that the maps were unbalanced between sexes.

## Discussion

Our method examined sex differences in homotopic co-activation and yielded a number of results, some of which were rather unexpected.

First, we found a sex bias in the Brainmap database, an observation that has sociological and metascientific relevance. In particular, there are more experiments that were performed only with male groups than with female groups. We also found a sex imbalance in the most studied cognitive domains. For example, females are more studied than males in the emotion domain, while males are more studied in the area of cognition. It is important to point out that our results do not necessarily mean that males are more commonly studied than females in fMRI research, as it remains theoretically possible that mixed-sex experiments, which were not the object of our study, enroll large numbers of female subjects. However, if the BrainMap database can be considered representative of the literature, it seems that biases come into play when conducting an fMRI experiment on a homogeneous sex group of subjects. On a practical level, this fact could have negative consequences for neuroscience research, such as producing an incomplete or distorted overview of human brain functioning. A consequence of the present work was that we were forced to use a complicated method to circumvent the problem.

The results of such methods tend to confirm the hypothesis that women show more interhemispheric co-activation than men, as suggested by the structural findings by Ingalhalikar et al. (2014). For example, most homotopic co-activation maps show significantly higher homotopic co-activations in women than in men. This would suggest that the female brain is less lateralized and more integrated than that of males, as it indicates that the two hemispheres tend to respond to task demands activating in tandem more often in women than men. On the other hand, as predicted, the results also draw a complex landscape and are not attributable to a simple and binary model (Joel 2021). In fact, Interception and Perception maps appear to be exceptions to the notion that women exhibit more interhemispheric integration than men. In the case of the Perception domain, women show significantly less average co-activation than men, while in the case of Interoception the difference is nominally in favor of men but not statistically significant.

Furthermore, we observed that in all maps, males have a higher number of significantly homotopically co-activated areas, although with low κ-values. This could potentially suggest that men show less (or rarer) intense but more extensive co-activations than women. This finding should not be the result of database biases, as our sampling procedures reduced the male dataset to the same size as the female one. Also, it might be feared that the more widespread co-activations of the male maps were just the effect of the higher spatial variability that may arise from sampling from a larger dataset. However, this seems not to be the case. In fact, the result of the one-sample t-tests should rule out that they were simply the result of a high variance since the t-test maps are virtually identical to those obtained with the average (Supplementary Figure S3). Furthermore, the higher number of voxels of each one of the 1000 male maps of the various domains compared to the female ones was confirmed by a series of two-sample t test. Nonetheless, due to the substantial differences between the female and male datasets, the effect of database bias cannot be completely ruled out.

Our results appear to confirm those of Zuo (Zuo et al. 2010) in reporting stronger HC for women in the posterior cingulate cortex and dorsolateral prefrontal cortex. Furthermore, the cluster in the posteromedial temporal lobe observed by Zuo as more correlated in males than in females falls into an area of greater male co-activation as we found in our results (Fig. 4). However, there are also a number of notable differences. In general, the areas of greatest difference in our results do not coincide with those indicated by Zuo (Zuo et al. 2010). For example, stronger female co-activation stands out in areas of social cognition such as the TPJ and the medial prefrontal cortex. Conversely, many primary perceptual and motor areas were found to be more co-activated in males. It would be difficult to explain these results on the basis of the sex imbalance of the cognitive subcategories. Although there is a higher percentage of social cognition studies in the female and male datasets, the difference seems too small to explain such a marked effect. Similarly, the greater proportion of male perception studies belonging to the ‘audition’ subdomain does not seem such as to justify the marked effect of male co-activation among bilateral STG. Curiously, this same area was found by Mancuso et al. (Mancuso et al. 2019b) as a region of significant difference between homotopic co-activation and resting state HC. Therefore, a possible explanation of the differences between our results and those of Zuo et al. could be given by the difference between HC at rest and co-activations during task. The level of convergence and divergence between co-activations and resting state connectivity is quite complex (Laird et al. 2013; Sepulcre et al. 2010; Cole et al. 2014; Torta et al. 2013)^.^ For instance, Goparaju et al. (Goparaju et al. 2014) suggested a rewiring of network paths during task execution in spite of overall maintained connectivity and hubness. And while Smith et al. (Smith et al. 2009) revealed a strong similarity between resting state and co-activation networks, it is important to consider that these networks are not identical.

We also observe a number of differences between the maps representing the specific cognitive domains. Unfortunately, it was not possible to perform any sampling within the domain-specific datasets to balance their cognitive fingerprint, due to the extremely small number of experiments belonging to some subdomains. So, some doubts remain about the reliability of these results. Nonetheless, the male and female datasets did not appear particularly unbalanced in this regard, showing an overall similar proportion of experiments (Fig. 2). This reassures us that domain-specific differences between sexes should not be primarily driven by database biases.

In addition, a number of repeating differences emerge across domains, which can be also observed in the domain-general map. TPJ, anterior insula, inferior frontal gyrus and, to a certain extent ventral mPFC show greater female co-activations; STG and sensorimotor areas show generally stronger co-activations in males. These observations might be supported by anatomic data. In fact, Björnholm and colleagues (Björnholm et al. 2017) reported that the fractional anisotropy of the corpus callosum is higher in males, while that of the splenium is higher in females. Since the body of the CC connects the motor and auditory areas, this could clarify the greater male homotopic co-activation of these regions. Partly in agreement with this, Genc and colleagues (Genc et al. 2018) report that women have more density than men in the splenium and genu, which connect the TPJ and frontal areas such as insula and IFG (Chao et al. 2009). Interestingly, our results show that these areas are more co-activated in women (Fig. 4). However, it should be pointed out that the presence of sex differences in the CC is a long-debated issue (Ardekani et al. 2013; Luders et al. 2014; Allen et al. 1991; Prendergast et al. 2015; Kurth et al. 2018; Vannucci et al. 2017; Lee et al. 2009). In addition, it should be considered that the callosal connections probably have both excitatory and inhibitory functions (Bloom and Hynd 2005), so it is not possible to identify a clear relationship between the CC structure and homotopic co-activations. On the other hand, Karolis and colleagues (2019) reported that cortical regions showing asymmetries in task-evoked activity have reduced connections with the opposite hemisphere, thus encouraging a comparison between our data and callosal connectivity.

Similarly, our results are in part consistent with some proposed psychological characterization of sexes. In the healthy population, men and women show differences in social cognition as well as in the social brain (Proverbio 2021). Women have greater emotional processing capacity, recognizing facial expressions of basic emotions more accurately and faster than men. Furthermore, women seem to engage more emotional brain areas during social cognition tasks (Navarra-Ventura et al. 2018). The fact that we found areas associated with social cognition such as TPJ showing stronger homotopic co-activation might be related to these observations.

## Limitation and future directions

A limitation of this study derives from the lack of balance in the number and type of experiments between the two sexes, which forced us to design a complex methodology to limit the bias. Despite our efforts, it cannot be excluded that this imbalance did not affect our results. In particular, the effect of larger co-activations in men could simply be due to the greater number of studies on the male population. Furthermore, while the domain-general datasets were counterbalanced in respect of their behavioral domains, the same was not possible at the subdomain level, as it would have produced too small samples of experiments. Therefore, the local differences between sex maps could be at least in part artefactual. We hope that, in the future, these disparities will be overcome and it will be possible to deal with more homogeneous databases.

A limitation of this study is that due to the meta-analytic nature of the data, it was not possible to assess handedness. Handedness has been shown to be related to functional connectivity (Tejavibulya et al. 2022), it is known to have a complex interplay with brain lateralization (Güntürkün and Ocklenburg 2017), in particular with that of language (Szaflarski et al. 2002). Left-handedness is also slightly more frequent in men (Papadatou-Paston et al. 2008), indicating an interaction between handedness, sex and interhemispheric connectivity. While we would expect the number of left-handed individuals in our database to be low, future studies might focus on the relation between these three variables.

A possible future development could be to evaluate the strength of interhemispheric co-activations in different domains in a large sample of female and male subjects, possibly relating it to the underlying functional and/or structural homotopic connectivity.

This study was based on the hypothesis that women have more homotopic co-activations due to greater integration of their connectome. However, it appears that sex differences in connectivity are actually an effect of brain volume (Hänggi et al. 2014; Martínez et al. 2017). For our analysis this is not particularly relevant: given that women tend to have smaller brains than men, it follows that they will tend to have greater interhemispheric integration and, based on our results, stronger homotopic co-activations. Nonetheless, future research will investigate the question and evaluate the effect of brain volume on HC and homotopic co-activation mechanisms.

## Conclusions

Our study provides empirical support for the hypothesis of greater interhemispheric integration in the female brain. However, there are considerable spatial differences in sex-specific differences in homotopic co-activation. In particular, it appears that primary motor and perceptual areas are more co-activated in males, in contrast to the general model. This argues for a multidimensional view of sex brain differences and suggests that the issue should be approached with more complex models than previously thought.

## Supplementary Information

Below is the link to the electronic supplementary material.

Supplementary file1 (DOCX 2444 KB)

## Data Availability

The data used in this study was obtained from BrainMap, a publicly available database (https://www.brainmap.org/). The resulting maps are available at https://figshare.com/projects/Gender_differences_in_Brain_Homotopic_Co-activations_a_meta-analytic_study/142511.

## References

[CR1] Aboitiz F, Montiel J (2003). One hundred million years of interhemispheric communication: the history of the corpus callosum. Braz J Med Biol Res.

[CR2] Agcaoglu O, Miller R, Mayer AR, Hugdahl K, Calhoun VD (2015). Lateralization of resting state networks and relationship to age and gender. Neuroimage.

[CR3] Allen LS, Richey MF, Chai YM, Gorski RA (1991). Sex differences in the corpus callosum of the living human being. J Neurosci.

[CR4] Ardekani BA, Figarsky K, Sidtis JJ (2013). Sexual dimorphism in the human corpus callosum: an MRI study using the OASIS brain database. Cereb Cortex.

[CR5] Björnholm L (2017). Structural properties of the human corpus callosum: Multimodal assessment and sex differences. Neuroimage.

[CR6] Bloom JS, Hynd GW (2005). The role of the corpus callosum in interhemispheric transfer of information: excitation or inhibition?. Neuropsychol Rev.

[CR7] Boles DB (2005). A large-sample study of sex differences in functional cerebral lateralization. J Clin Exp Neuropsychol.

[CR8] Botvinik-Nezer R, Holzmeister F, Camerer CF (2020). Variability in the analysis of a single neuroimaging dataset by many teams. Nature.

[CR9] Bruner E, de la Cuétara JM, Colom R, Martin-Loeches M (2012). Gender-based differences in the shape of the human corpus callosum are associated with allometric variations. J Anat.

[CR10] Byne W, Bleier R, Houston L (1988). Variations in human corpus callosum do not predict gender: a study using magnetic resonance imaging. Behav Neurosci.

[CR11] Cauda F (2020). Hubs of long-distance co-alteration characterize brain pathology. Hum Brain Mapp.

[CR12] Cauda F (2021). Interhemispheric co-alteration of brain homotopic regions. Brain Struct Funct.

[CR13] Chao YP (2009). Probabilistic topography of human corpus callosum using cytoarchitectural parcellation and high angular resolution diffusion imaging tractography. Hum Brain Mapp.

[CR14] Chen J (2016). Long-term acclimatization to high-altitude hypoxia modifies interhemispheric functional and structural connectivity in the adult brain. Brain Behav.

[CR15] Cole MW, Bassett DS, Power JD, Braver TS, Petersen SE (2014). Intrinsic and task-evoked network architectures of the human brain. Neuron.

[CR16] Costa T (2021). BACON: A tool for reverse inference in brain activation and alteration. Hum Brain Mapp.

[CR17] Del Giudice M (2021). Binary thinking about the sex binary: a comment on Joel. Neurosci Biobehav Rev.

[CR18] Deng K (2021). Impaired robust interhemispheric function integration of depressive brain from REST-meta-MDD database in China. Bipolar Disord.

[CR19] Donishi T, Terada M, Kaneoke Y (2017). Effects of gender, digit ratio, and menstrual cycle on intrinsic brain functional connectivity: a whole-brain, voxel-wise exploratory study using simultaneous local and global functional connectivity mapping. Brain Behav.

[CR20] Eickhoff SB (2009). Coordinate-based activation likelihood estimation meta-analysis of neuroimaging data: a random-effects approach based on empirical estimates of spatial uncertainty. Hum Brain Mapp.

[CR21] Eickhoff SB, Bzdok D, Laird AR, Kurth F, Fox PT (2012). Activation likelihood estimation meta-analysis revisited. Neuroimage.

[CR22] Fernández G (2003). Menstrual cycle-dependent neural plasticity in the adult human brain is hormone, task, and region specific. J Neurosci.

[CR23] Fox PT, Lancaster JL (2002). Mapping context and content: The BrainMap model. Nat Rev Neurosci.

[CR24] Fox MD (2005). The human brain is intrinsically organized into dynamic, anticorrelated functional networks. Proc Natl Acad Sci USA.

[CR25] Fox PT (2005). BrainMap taxonomy of experimental design: description and evaluation. Human Brain Mapp.

[CR26] Friedrich P, Ocklenburg S, Heins N, Schlüter C, Fraenz C, Beste C, Güntürkün O, Genç E (2017). Callosal microstructure affects the timing of electrophysiological left-right differences. Neuroimage.

[CR27] Friston K (2002). Functional integration and inference in the brain. Prog Neurobiol.

[CR28] Genc S, Malpas CB, Ball G, Silk TJ, Seal ML (2018). Age, sex, and puberty related development of the corpus callosum: a multi-technique diffusion MRI study. Brain Struct Funct.

[CR29] Goldstein, A., Covington, B. P., Mahabadi, N. & Mesfin, F. B. (2021) Neuroanatomy, corpus callosum. In: *StatPearls*. Treasure Island (FL): StatPearls Publishing 28846239

[CR30] Goparaju B, Rana KD, Calabro FJ, Vaina LM (2014). A computational study of whole-brain connectivity in resting state and task fMRI. Med Sci Monit.

[CR31] Gotts SJ (2013). Two distinct forms of functional lateralization in the human brain. Proc Natl Acad Sci USA.

[CR32] Grabowska A (2017). Sex on the brain: Are gender-dependent structural and functional differences associated with behavior?. J Neurosci Res.

[CR33] Gracia-Tabuenca Z, Moreno MB, Barrios FA, Alcauter S (2018). Hemispheric asymmetry and homotopy of resting state functional connectivity correlate with visuospatial abilities in school-age children. Neuroimage.

[CR34] Güntürkün O, Ocklenburg S (2017). Ontogenesis of lateralization. Neuron.

[CR35] Guo L, Zhou F, Zhang N, Kuang H, Feng Z (2019). Frequency-specific abnormalities of functional homotopy in alcohol dependence: a resting-state functional magnetic resonance imaging study. Neuropsychiatr Dis Treat.

[CR36] Habib M (1991). Effects of handedness and sex on the morphology of the corpus callosum: a study with brain magnetic resonance imaging. Brain Cogn.

[CR37] Hänggi J, Fövenyi L, Liem F, Meyer M, Jäncke L (2014). The hypothesis of neuronal interconnectivity as a function of brain size-a general organization principle of the human connectome. Front Hum Neurosci.

[CR38] Hausmann M (2005). Hemispheric asymmetry in spatial attention across the menstrual cycle. Neuropsychologia.

[CR39] Hausmann M, Becker C, Gather U, Güntürkün O (2002). Functional cerebral asymmetries during the menstrual cycle: a cross-sectional and longitudinal analysis. Neuropsychologia.

[CR40] Hirnstein M, Hausmann M (2021). Sex/gender differences in the brain are not trivial-A commentary on Eliot et al. Neurosci Biobehav Rev.

[CR41] Hiscock M, Israelian M, Inch R, Jacek C, Hiscock-kalil C (1995). Is there a sex difference in human laterality? II. An exhaustive survey of visual laterality studies from six neuropsychology journals. J Clin Exp Neuropsychol.

[CR42] Hjelmervik H (2012). Language lateralization and cognitive control across the menstrual cycle assessed with a dichotic-listening paradigm. Psychoneuroendocrinology.

[CR43] Hjelmervik H, Hausmann M, Osnes B, Westerhausen R, Specht K (2014). Resting states are resting traits–an FMRI study of sex differences and menstrual cycle effects in resting state cognitive control networks. PLoS ONE.

[CR44] Ingalhalikar M (2014). Sex differences in the structural connectome of the human brain. Proc Natl Acad Sci USA.

[CR45] Jager G, Postma A (2003). On the hemispheric specialization for categorical and coordinate spatial relations: a review of the current evidence. Neuropsychologia.

[CR46] Jin X, Liang X, Gong G (2020). Functional integration between the two brain hemispheres: evidence from the homotopic functional connectivity under resting state. Front Neurosci.

[CR47] Joel D (2021). Beyond the binary: Rethinking sex and the brain. Neurosci Biobehav Rev.

[CR48] Joel D (2015). Sex beyond the genitalia: the human brain mosaic. Proc Natl Acad Sci USA.

[CR49] Joel D (2018). Analysis of human brain structure reveals that the brain “types” typical of males are also typical of females, and vice versa. Front Hum Neurosci.

[CR50] Joel D, Garcia-Falgueras A, Swaab D (2020). The complex relationships between sex and the brain. Neuroscientist.

[CR51] Kanaan RA (2012). Gender differences in white matter microstructure. PLoS ONE.

[CR52] Karolis VR, Corbetta M, Thiebaut de Schotten M (2019). The architecture of functional lateralisation and its relationship to callosal connectivity in the human brain. Nat Commun.

[CR53] Kurth F, Spencer D, Hines M, Luders E (2018). Sex differences in associations between spatial ability and corpus callosum morphology. J Neurosci Res.

[CR54] Laird AR, Lancaster JL, Fox PT (2005). BrainMap: The social evolution of a human brain mapping database. Neuroinformatics.

[CR55] Laird AR (2011). Behavioral interpretations of intrinsic connectivity networks. J Cogn Neurosci.

[CR56] Laird AR (2013). Networks of task co-activations. Neuroimage.

[CR57] Lancaster JL (1997). Automated labeling of the human brain: a preliminary report on the development and evaluation of a forward-transform method. Hum Brain Mapp.

[CR58] Lancaster JL (2000). Automated Talairach Atlas labels for functional brain mapping. Hum Brain Mapp.

[CR59] Lee BY (2009). A volumetric study of the corpus callosum in 20s and 40s Korean people. Brain Struct Funct.

[CR60] Li HJ, Xu Y, Zhang KR, Hoptman MJ, Zuo XN (2015). Homotopic connectivity in drug-naïve, first-episode, early-onset schizophrenia. J Child Psychol Psychiatry.

[CR61] Liu H, Stufflebeam SM, Sepulcre J, Hedden T, Buckner RL (2009). Evidence from intrinsic activity that asymmetry of the human brain is controlled by multiple factors. Proc Natl Acad Sci USA.

[CR62] Loomba N, Beckerson ME, Ammons CJ, Maximo JO, Kana RK (2021). Corpus callosum size and homotopic connectivity in autism spectrum disorder. Psychiatry Res Neuroimaging.

[CR63] Luders E, Toga AW, Thompson PM (2014). Why size matters: differences in brain volume account for apparent sex differences in callosal anatomy: the sexual dimorphism of the corpus callosum. Neuroimage.

[CR64] Mancuso L, Uddin LQ, Nani A, Costa T, Cauda F (2019). Brain functional connectivity in individuals with callosotomy and agenesis of the corpus callosum: a systematic review. Neurosci Biobehav Rev.

[CR65] Mancuso L (2019). The homotopic connectivity of the functional brain: a meta-analytic approach. Sci Rep.

[CR66] Manuello J, Nani A, Premi E, Borroni B, Costa T, Tatu K, Liloia D, Duca S, Cauda F (2018). The pathoconnectivity profile of Alzheimer's Disease: a morphometric coalteration network analysis. Front Neurol.

[CR67] Manuello J, Costa T, Cauda F, Liloia D (2022). Six actions to improve detection of critical features for neuroimaging coordinate-based meta-analysis preparation. Neurosci Biobehav Rev.

[CR68] Martínez K (2017). Individual differences in the dominance of interhemispheric connections predict cognitive ability beyond sex and brain size. Neuroimage.

[CR69] McCarthy MM (2016). Multifaceted origins of sex differences in the brain. Philos Trans R Soc Lond B Biol Sci.

[CR70] McGlone J (1980). Sex differences in human brain asymmetry: a critical survey. Behav Brain Sci.

[CR71] Mollink J (2019). The spatial correspondence and genetic influence of interhemispheric connectivity with white matter microstructure. Nat Neurosci.

[CR72] Müller VI (2018). Ten simple rules for neuroimaging meta-analysis. Neurosci Biobehav Rev.

[CR73] Navarra-Ventura G (2018). Gender differences in social cognition: a cross-sectional pilot study of recently diagnosed patients with Schizophrenia and healthy subjects. Can J Psychiatry.

[CR74] Nielsen JA, Zielinski BA, Ferguson MA, Lainhart JE, Anderson JS (2013). An evaluation of the left-brain vs. right-brain hypothesis with resting state functional connectivity magnetic resonance imaging. PLoS ONE.

[CR75] Page MJ (2021). The PRISMA 2020 statement: an updated guideline for reporting systematic reviews. BMJ.

[CR76] Papadatou-Pastou M, Martin M, Munafò MR, Jones GV (2008). Sex differences in left-handedness: a meta-analysis of 144 studies. Psychol Bull.

[CR77] Patel RS, Bowman FDB, Rilling JK (2006). A Bayesian approach to determining connectivity of the human brain. Hum Brain Mapp.

[CR78] Pletzer B, Jäger S, Hawelka S (2019). Sex hormones and number processing. Progesterone and testosterone relate to hemispheric asymmetries during number comparison. Horm Behav.

[CR79] Prendergast DM (2015). Age and sex effects on corpus callosum morphology across the lifespan. Hum Brain Mapp.

[CR80] Pritschet L (2020). Functional reorganization of brain networks across the human menstrual cycle. Neuroimage.

[CR81] Proverbio AM (2021). Sex differences in the social brain and in social cognition. J Neurosci Res.

[CR82] Salminen LE (2021). Sex is a defining feature of neuroimaging phenotypes in major brain disorders. Hum Brain Mapp.

[CR83] Schaefer A (2018). Local-Global parcellation of the human cerebral cortex from intrinsic functional connectivity MRI. Cereb Cortex.

[CR84] Schmied A (2020). Sex differences associated with corpus callosum development in human infants: a longitudinal multimodal imaging study. Neuroimage.

[CR85] Sepulcre J (2010). The organization of local and distant functional connectivity in the human brain. PLoS Comput Biol.

[CR86] Shan X (2021). Shared and distinct homotopic connectivity changes in melancholic and non-melancholic depression. J Affect Disord.

[CR87] Shiino A (2017). Sex-related difference in human white matter volumes studied: Inspection of the corpus callosum and other white matter by VBM. Sci. Reports..

[CR88] Smith SM (2009). Correspondence of the brain’s functional architecture during activation and rest. Proc Natl Acad Sci USA.

[CR89] Sporns O (2013). Network attributes for segregation and integration in the human brain. Curr Opin Neurobiol.

[CR90] Stark DE (2008). Regional variation in interhemispheric coordination of intrinsic hemodynamic fluctuations. J Neurosci.

[CR91] Suganthy J (2003). Gender- and age-related differences in the morphology of the corpus callosum. Clin Anat.

[CR92] Szaflarski JP (2002). Language lateralization in left-handed and ambidextrous people: fMRI data. Neurology.

[CR93] Tejavibulya L (2022). Large-scale differences in functional organization of left- and right-handed individuals using whole-brain, data-driven analysis of connectivity. Neuroimage.

[CR94] Tomasi D, Volkow ND (2012). Laterality patterns of brain functional connectivity: Gender effects. Cereb Cortex.

[CR95] Toro R, Fox PT, Paus T (2008). Functional co-activation map of the human brain. Cereb Cortex.

[CR96] Torta DM, Costa T, Duca S, Fox PT, Cauda F (2013). Parcellation of the cingulate cortex at rest and during tasks: a meta-analytic clustering and experimental study. Front Hum Neurosci.

[CR97] Turkeltaub PE (2012). Minimizing within-experiment and within-group effects in activation likelihood estimation meta-analyses. Hum Brain Mapp.

[CR98] Vallesi A (2021). Fronto-parietal homotopy in resting-state functional connectivity predicts task-switching performance. Brain Struct Funct.

[CR99] van den Heuvel MP, Sporns O (2013). An anatomical substrate for integration among functional networks in human cortex. J Neurosci.

[CR100] Vannucci RC, Barron TF, Vannucci SJ (2017). Development of the corpus callosum: an MRI study. Dev Neurosci.

[CR101] Voyer D, Voyer S, Bryden MP (1995). Magnitude of sex differences in spatial abilities: A meta-analysis and consideration of critical variables. Psychol Bull.

[CR102] Wendt PE, Risberg J (1994). Cortical activation during visual spatial processing: relation between hemispheric asymmetry of blood flow and performance. Brain Cogn.

[CR103] Wiersch L, Weis S (2021). Sex differences in the brain: more than just male or female. Cogn Neurosci.

[CR104] Williams CM, Peyre H, Toro R, Ramus F (2021). Sex differences in the brain are not reduced to differences in body size. Neurosci Biobehav Rev.

[CR105] Yao S (2021). Decreased homotopic interhemispheric functional connectivity in children with autism spectrum disorder. Autism Res.

[CR106] Yu D (2018). Altered interhemispheric resting-state functional connectivity in young male smokers. Addict Biol.

[CR107] Zhang Y (2021). The human brain is best described as being on a female/male continuum: evidence from a neuroimaging connectivity study. Cereb Cortex.

[CR108] Zhao L (2017). Altered interhemispheric functional connectivity in remitted bipolar disorder: A Resting State fMRI Study. Sci Rep.

[CR109] Zhao J (2020). Age-related decreases in interhemispheric resting-state functional connectivity and their relationship with executive function. Front Aging Neurosci.

[CR110] Zuo XN (2010). Growing together and growing apart: regional and sex differences in the lifespan developmental trajectories of functional homotopy. J Neurosci.

