# Supplementary material for: Sex differences in brain homotopic co-activations: a meta-analytic study
Source: Brain Struct Funct. 2022 Oct 21;227(8):2839–55. doi: 10.1007/s00429-022-02572-0 (PMC9618505; doi:10.1007/s00429-022-02572-0)
Supplement: Supplementary file 1 — Supplementary file1 (DOCX 2444 KB) [file 429_2022_2572_MOESM1_ESM.docx]

Sex differences in Brain Homotopic Co-activations:

a meta-analytic study

Supplementary Materials

Chiara Bonelli^1*^, Lorenzo Mancuso^1*^, Jordi Manuello^1,2^, Donato Liloia^1,2^, Tommaso Costa^1,2^, Franco Cauda^1,2^

^1^FocusLab, Department of Psychology, University of Turin, Turin, Italy

^2^GCS-fMRI, Koelliker Hospital and Department of Psychology, University of Turin, Turin, Italy

*These authors contributed equally to the work


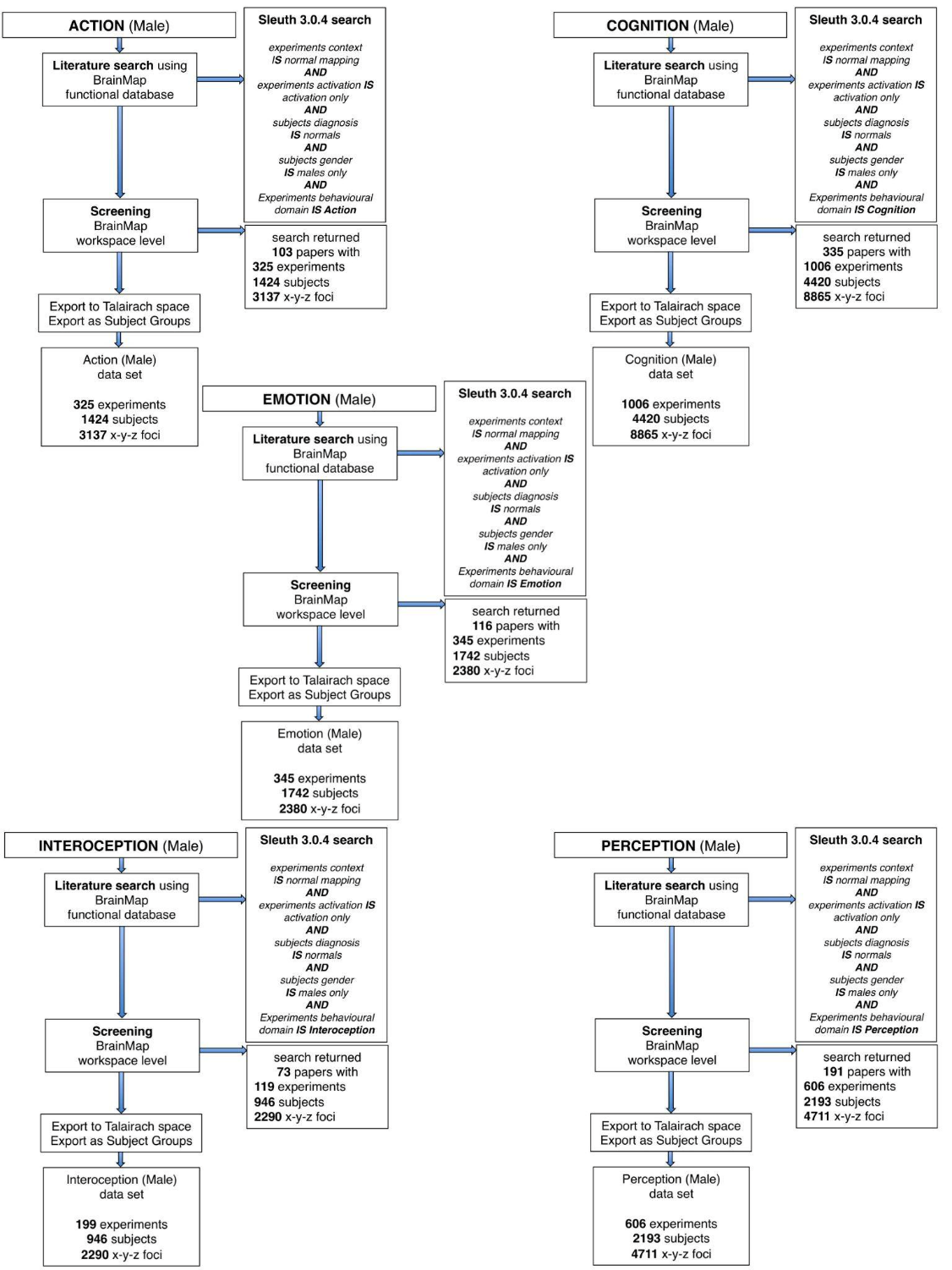


###### **Figure S1.** PRISMA flow chart - Male


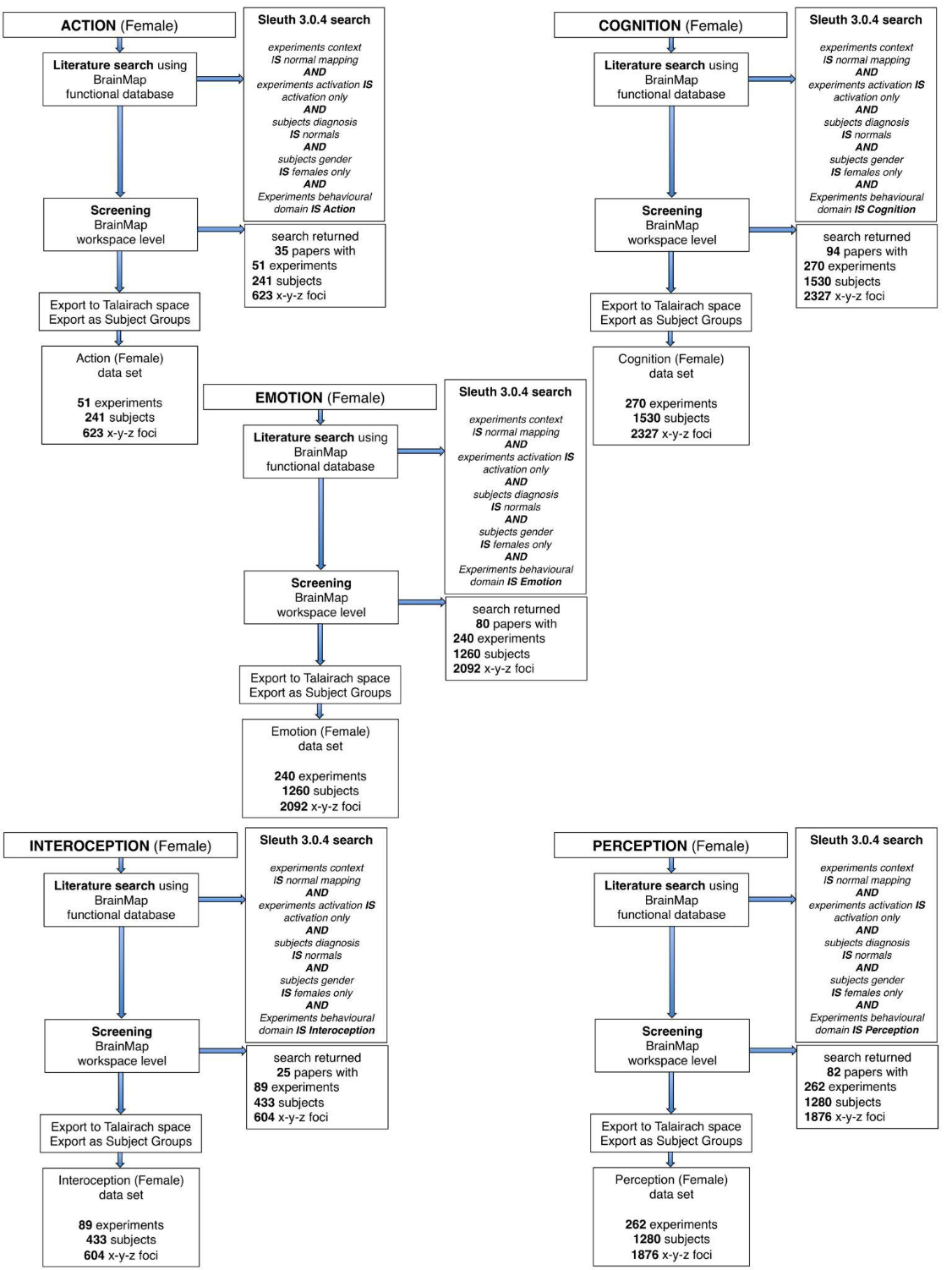


###### **Figure S2.** PRISMA flow chart - Female


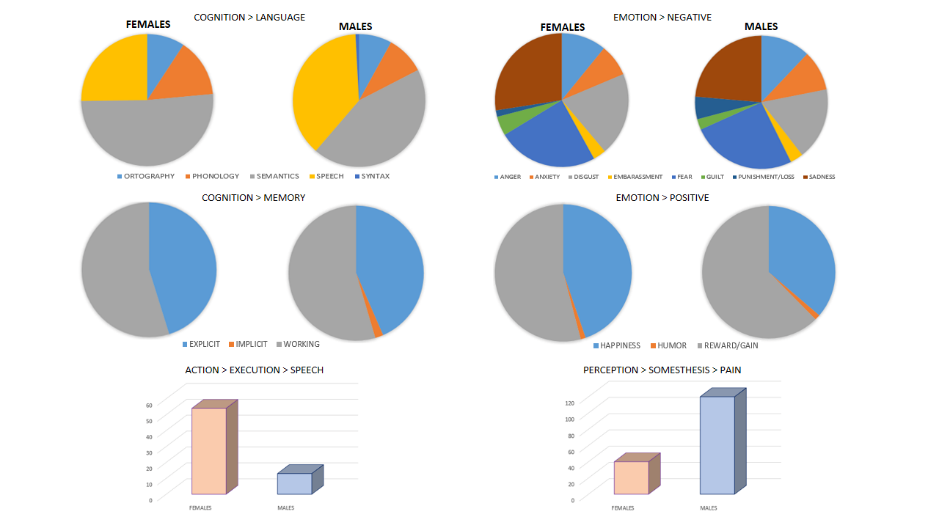


###### **Figure S3.** Proportions of investigated cognitive sub-domains by sex


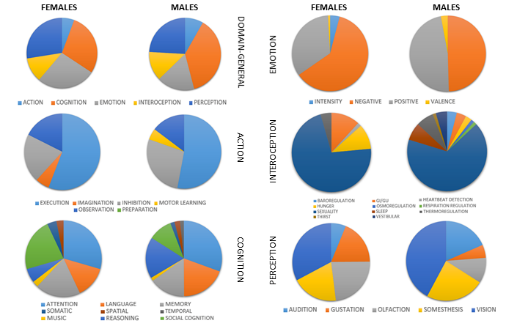


**Figure S4.** Proportions of investigated cognitive domains and sub-domains by sex in the post-2005 experiments.


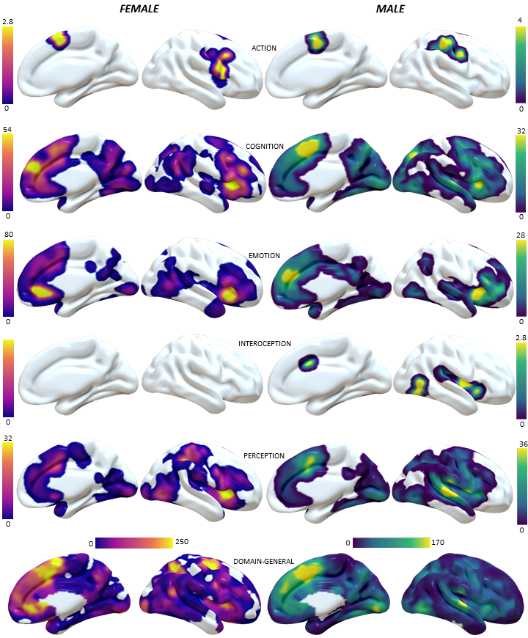


**Figure S5.** Surface mapping of the one-sample t-test MHC maps for the two sexes and the different domains


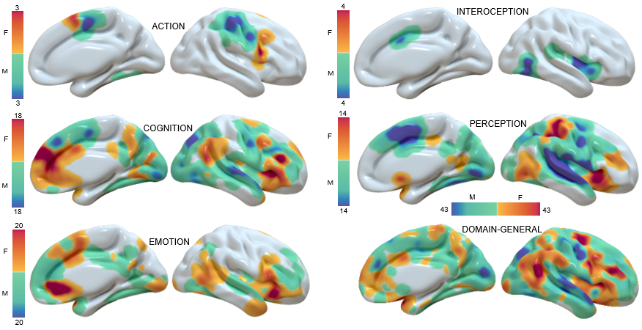


**Figure S6.** Surface mapping of the two-sample t-tests between the two sexes for the different domains. Warm color voxels are significant in the female > males contrast, cold color voxels are significant in the males > females contrast.

**Further tests on the female/male ratios**

**Methods**

We wanted to further verify if the female maps had less widespread but overall stronger homotopic co-activations than these of men. To do so, we counted the number of non-zero voxels in the maps of the two sexes for a given domain, and calculated the mean of their values. We then obtained the *female* / *male* ratio of these two parameters. Values < 1 indicate that the female map is lower than the male one on that parameter, values > 1 indicate the opposite. To test if those values were significantly ≠ 1, we proceeded as follows. First, for a given cognitive domain, 500 MHC maps were randomly extracted from the 1000 maps sample used to build the final female map, and another 500 from the male sample. They were then averaged in a map that represented the HC of a sort of a “sex neutral” brain in that domain. Another sex neutral map was produced with the same method, and the number of non-zero voxels and the average of their values were calculated in both maps, to produce their respective ratios. Then, this procedure was repeated 1000 times, to produce a null distribution for each of these two ratios. The mean of these distributions approximate 1, and their standard deviation models the random variability of a couple of MHC maps built with the same data of the real maps but deprived of any sex specificity. Two two-tailed t-tests were thus performed, testing that the original ratios were different from one. The only ratio to be not significantly ≠ 1 at any threshold was the one between the average values of non-zero voxels of the two sexes in the Interoception domain (italics in Table 1). All the other ratios were significantly different from 1 with *p* ≪ .001.

###### **Table S1.** Number of non-zero voxels and average value of non-zero voxels for each of the maps presented in Figure 2, and the ratio between female maps and male maps. Ratios > 1 indicate that the female map has a larger value than that of men, ratios < 1 indicate the opposite.

| **Cognitive Domain** | **N. non-zero voxels** | | | | **Average value of non-zero voxels** | | | |
| --- | --- | --- | --- | --- | --- | --- | --- | --- |
|  | **F** | **M** | **F/M** | ***p*** | **F** | **M** | **F/M** | ***p*** |
| Domain-general | 77226 | 103836 | 0.74 | ≪ .001 | 0.29 | 0.24 | 1.19 | ≪ .001 |
| Action | 2654 | 13328 | 0.2 | ≪ .001 | 0.02 | 0.01 | 4.33 | ≪ .001 |
| Cognition | 41370 | 85480 | 0.48 | ≪ .001 | 0.14 | 0.07 | 1.95 | ≪ .001 |
| Emotion | 30704 | 43694 | 0.7 | ≪ .001 | 0.11 | 0.05 | 2.14 | ≪ .001 |
| Interoception | 1138 | 14084 | 0.08 | ≪ .001 | .0039 | .0041 | 0.93 | 0.28 |
| Perception | 39278 | 65384 | 0.6 | ≪ .001 | 0.07 | 0.08 | 0.87 | ≪ .001 |

**Table S2.** Contingency table with the number of areas more activated in the female or male maps for each domain.

|  | **F** | **M** | total |
| --- | --- | --- | --- |
| **Domain-general** | 139 | 247 | 386 |
| **Action** | 7 | 53 | 60 |
| **Cognition** | 91 | 226 | 317 |
| **Emotion** | 67 | 115 | 182 |
| **Interoception** | 3 | 48 | 51 |
| **Perception** | 78 | 188 | 266 |
| total | 385 | 877 | 1262 |
